# Dexamethasone-Enhanced Microdialysis and Penetration Injury

**DOI:** 10.3389/fbioe.2020.602266

**Published:** 2020-12-08

**Authors:** Andrea Jaquins-Gerstl, Adrian C. Michael

**Affiliations:** Department of Chemistry, University of Pittsburgh, Pittsburgh, PA, United States

**Keywords:** microdialysis, dexamethasone, penetration injury, brain, mitigate

## Abstract

Microdialysis probes, electrochemical microsensors, and neural prosthetics are often used for *in vivo* monitoring, but these are invasive devices that are implanted directly into brain tissue. Although the selectivity, sensitivity, and temporal resolution of these devices have been characterized in detail, less attention has been paid to the impact of the trauma they inflict on the tissue or the effect of any such trauma on the outcome of the measurements they are used to perform. Factors affecting brain tissue reaction to the implanted devices include: the mechanical trauma during insertion, the foreign body response, implantation method, and physical properties of the device (size, shape, and surface characteristics. Modulation of the immune response is an important step toward making these devices with reliable long-term performance. Local release of anti-inflammatory agents such as dexamethasone (DEX) are often used to mitigate the foreign body response. In this article microdialysis is used to locally deliver DEX to the surrounding brain tissue. This work discusses the immune response resulting from microdialysis probe implantation. We briefly review the principles of microdialysis and the applications of DEX with microdialysis in (i) neuronal devices, (ii) dopamine and fast scan cyclic voltammetry, (iii) the attenuation of microglial cells, (iv) macrophage polarization states, and (v) spreading depolarizations. The difficulties and complexities in these applications are herein discussed.

## Introduction

Microdialysis is a powerful technique for near real-time intracranial chemical monitoring in both animal models and human patients (Roberts and Anderson, 1979; Ungerstedt, 1984, 1991; Benveniste et al., 1987, 1989; Benveniste, 1989; Santiago and Westerink, 1990; Carneheim and Stahle, 1991; Parsons et al., 1991; Dykstra et al., 1992; Stenken, 1999; Hutchinson et al., 2000; Chefer et al., 2001; Stenken et al., 2001, 2010; Bosche et al., 2003, 2010; Schuck et al., 2004; Ungerstedt and Rostami, 2004; Parkin et al., 2005; Shou et al., 2006; Mitala et al., 2008; Hashemi et al., 2009; Jaquins-Gerstl et al., 2011; Wang and Michael, 2012; Zhang et al., 2013; Nesbitt et al., 2015; Dreier et al., 2016). A key advantage of microdialysis is its broad scope: it has been used to monitor neurotransmitters, peptides, amino acids, metabolites, and hormones, etc. This is possible because the only requirement is that the molecule of interest be smaller than the molecular weight cutoff of the chosen membrane material. As probes are perfused with, typically, an artificial cerebrospinal fluid solution (aCSF), small molecules enter the probe by passive diffusion and are swept to the probe outlet. The outlet is either interfaced directly to an analytical system, such as liquid chromatography, capillary electrophoresis, or mass spectrometry for on-line analysis, or discrete dialysate samples are collected and stored for later off-line analysis. Because microdialysis is highly compatible with awake-behaving animals, numerous studies have examined the relationships between chemical events in the extracellular space of the brain and animal behaviors (Becker and Cha, 1989; Robinson and Justice, 1991; Castner et al., 1993; Borjigin and Liu, 2008). Microdialysis also finds use in anesthetized or sedated patients receiving intensive care, after traumatic brain injury for example (Globus et al., 1995; Strong et al., 2002, 2005, 2007; Ungerstedt and Rostami, 2004; Westerink and Cremers, 2007; Haddad and Arabi, 2012; Sanchez et al., 2013; Rogers et al., 2017; Booth et al., 2018; Pagkalos et al., 2018; Gowers et al., 2019).

Slow temporal resolution is often mentioned as a limitation of microdialysis. It is important, however, to consider the factors that limit temporal resolution. Often, extended sample collection times are necessary to assure that a detectable quantity of target analyte is available. Thus, over time, various workers have improved the temporal resolution of microdialysis by developing refined analytical methods with lower detection limits. For example, the Kennedy group has developed numerous rapid dialysate analyses by on-line capillary liquid chromatography or on-line capillary electrophoresis (Watson et al., 2006; Li et al., 2009; Perry et al., 2009; Wang et al., 2010; Lee et al., 2013; Kennedy et al., 2018; Ngernsutivorakul et al., 2018). The Andrews and Weber groups have used capillary liquid chromatography to analyze dopamine and serotonin in brain dialysates with minute and sub-minute temporal resolution (Liu et al., 2010; Yang et al., 2013; Zhang et al., 2013; Gu et al., 2015; Wilson and Michael, 2017).

Past observations from our laboratory have suggested, further, that an additional limitation of the temporal resolution of microdialysis-based monitoring stems from the so-called penetration trauma of the tissue surrounding the track of the microdialysis probe itself. Early experiments in the Michael lab attempted to monitor electrically evoked dopamine release in the rat striatum using a dopamine-sensitive microelectrode interfaced to the outlet line of a microdialysis probe (Lu et al., 1998; Yang and Michael, 2007; Nesbitt et al., 2013; Varner et al., 2016). *In vitro* characterization of the probe-microelectrode combination suggested a temporal resolution near 30 s, sufficiently rapid to monitor evoked dopamine responses recorded at microelectrodes implanted directly in brain tissue. However, evoked responses were not observable at the probe outlet without the aid of a dopamine uptake inhibitor, nomifensine (Borland et al., 2005). Eventually, we performed experiments with microelectrodes implanted side-by-side with microdialysis probes: without the aid of the uptake inhibitor there was, unexpectedly, no evoked response in the tissue surrounding the probe. Thus, our failure to detect evoked dopamine release at the probe outlet was not a matter of temporal resolution but rather was a matter of tissue disruption: there was no evoked response taking place in the surrounding tissue (Borland et al., 2005). This finding raised our initial concerns that the tissue surrounding the probe was in an abnormal, most likely traumatized, state and prompted our subsequent focus on the issue of penetration trauma and, eventually, strategies to mitigate it. These strategies are the focus of this review article.

A key, and somewhat unique, strategy available to the mitigation of penetration trauma induced by the implantation of microdialysis probes is so-called “retrodialysis” (Stenken, 1999; Shippenberg and Thompson, 2001; Cano-Cebrian et al., 2005). Retrodialysis is the term coined for the delivery of substances to brain tissues via the microdialysis probe itself. The focus of this review is our experience with dexamethasone (DEX)-enhanced microdialysis, which involves the retrodialysis of DEX to the probe track (Schuck et al., 2004; Nesbitt, 2015; Nesbitt et al., 2015; Kozai et al., 2016). DEX is a very well-known anti-inflammatory agent that has proven, thus far, highly effective at mitigating probe-induced penetration trauma during intracranial microdialysis in both the rat striatum and the rat cortex.

Dexamethasone-enhanced microdialysis offers key and specific benefits. First, it has facilitated the detection of evoked dopamine release at the probe outlet, resolving the technical difficulty described above. Second, we have found that DEX-enhanced microdialysis offers stabilized monitoring performance in brain tissue for at least 10 days following probe insertion. We hope that this improvement in longitudinal monitoring capability will be a benefit especially to patients who require neuromonitoring after traumatic brain injury.

Of course, DEX is not just an anti-inflammatory agent: it is steroid with its own potential neurochemical effects, raising the possibility that it might alter the outcome of neurochemical investigations. This, of course, must be acknowledged and addressed. As will be discussed later in this review, we have found that it is not necessary to deliver DEX continuously throughout an extended period of microdialysis monitoring. Preliminary evidence suggests that DEX retrodialysis is mainly needed at the time of probe insertion and that its benefits last beyond the termination of retrodialysis (Nesbitt, 2015; Nesbitt et al., 2015; Kozai et al., 2016; Varner et al., 2016). This is a matter that potential adopters of DEX-enhanced microdialysis will need to evaluate in the future in the context of their own particular intended applications.

## Principles of Microdialysis

The underlying process driving microdialysis sampling is the passive diffusion of substances across a semi-permeable hollow-fiber dialysis membrane (Stenken et al., 1997, 2001, 2010; Stenken, 1999; Chefer et al., 2001; Bungay et al., 2007; Chaurasia et al., 2007; Huang et al., 2008; Darvesh et al., 2011). Various polymeric materials, with molecular weight cutoff values between 10 and 100 kDa, are available for probe construction (Stenken, 1999; Chefer et al., 2001; Bungay et al., 2007; Darvesh et al., 2011; Hammarlund-Udenaes, 2017). The overall sampling efficiency, however, is influenced by the membrane and the tortuosity and volume fraction of the brain tissue, the tendency of analyte molecules to stick to the membrane or connecting tubing, the stability of the analyte molecule, etc. (Torto et al., 1999). The probes are commonly between 200 and 500 μm in external diameter and several millimeters in length, chosen to match the target brain structure. The dialysate concentration of a target analyte is proportional, but not quantitatively equal, to its concentration in the surrounding extracellular space (Ungerstedt, 1991). The analyte concentration at the probe outlet (C_OUT_) reflects two contributions, one derived from the external medium (C_EXT_), and one derived from the probe inlet (C_IN_) if analyte retrodialysis is ongoing. The relationship between these quantities is:

(1) $${\text{C}}_{\text{OUT}}=\left(1-\text{E}\right)\,{\text{C}}_{\text{IN}}+{\text{RC}}_{\text{EXT},\infty }$$

where E is the extraction fraction and *R* is the so-called relative recovery: the term C_*EXT*,∞_ denotes the concentration of analyte in the external medium sufficiently far from the probe so as not to be disturbed by the probe *per se*. “Conventional microdialysis” refers to the case that C_*IN*_ = 0. A common rearrangement of Eq. 1 leads to the concentration differences plot:

(2) $${\text{C}}_{\text{IN}}-{\text{C}}_{\text{OUT}}={\text{EC}}_{\text{IN}}-{\text{RC}}_{\text{EXT},\infty }$$

Equation 2 shows that a plot of C_IN_–C_OUT_ (the concentration differences) against C_IN_ is expected to be a straight line, with a slope of E and an x-intercept of RC_EXT,∞_. While such a plot provides the value of the extraction fraction, the relative recovery cannot generally be measured without independent knowledge of the external concentration. Thus, while *in vitro* probe calibration is straightforward, generally the value of *R* is an uncertain quantity during *in vivo* measurements. It is now generally recognized that *R* values determined during *in vitro* calibration are not reliably applicable to *in vivo* conditions: investigators need to keep this in mind. Our preference, and recommendation, is to report “dialysate concentrations” of analytes of interest, without attempting to convert to *in vivo* concentrations. A number of mathematical models have been developed in an effort to theoretically predict *in vivo R* values (Amberg and Lindefors, 1989; Benveniste, 1989; Lindefors et al., 1989; Bungay et al., 1990, 2003, 2007) but such work is beyond the scope of this review.

## Inflammation

### Penetration Trauma

**FIGURE 1:**
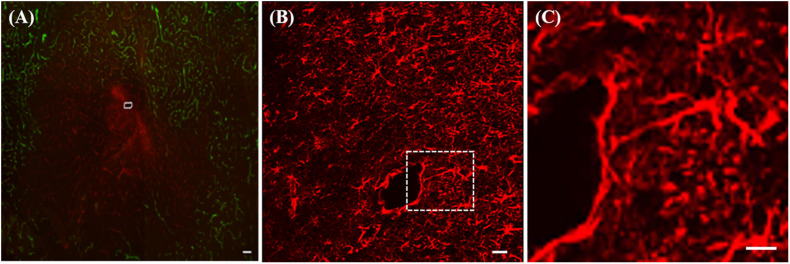
**(A)** Fluorescence image of ischemia (green-blood vessels) and a PECAM (red) halo at a microdialysis probe track (white marking) in rat striatum. **(B)** GFAP immunoreactivity after 24-h probe implant, **(C)** enlargement of the area in the white box in B showing a glial cell extending a process ∼300 μm toward the track. All scale bars = 100 μM. Adopted from references (Mitala et al., 2008; Jaquins-Gerstl and Michael, 2009).

The average spacing of the microvessels in brain tissues is around 50 μm and a number of larger blood vessels are also present. Inevitably, therefore, insertion of microdialysis probes and other neural devices such as microelectrode arrays with dimensions greater than 50 μm induces penetration trauma (Benveniste and Diemer, 1987; Zhou et al., 2001). In one of our first studies using immunohistochemistry we labeled blood vessels with dye-laden polystyrene nanobeads (100 nm in diameter) delivered to the brain by transcardial perfusion and the antibody platelet endothelial cell adhesion molecule (PECAM), a histochemical marker for endothelial cells. In healthy tissue the blood vessels were double-labeled with nanobeads and PECAM. However, tissue near the microdialysis probe tracks exhibited ischemia (diminished blood flow), in the form of PECAM immunoreactivity and blood vessels devoid of nanobeads, Figure 1A (Mitala et al., 2008). Probe tracks were surrounded by endothelial cell debris, which appeared as a diffuse halo of PECAM immunoreactivity and there was a large region that was devoid of the nanobeads indicating a lack of blood flow (Jaquins-Gerstl and Michael, 2009).

Moreover, the penetration trauma triggers a tissue response: sometimes called a foreign body response or a wound-healing response. The tissue response, left unattended, leads to gliosis of the probe track. Within a few days of insertion, microdialysis probes are surrounded by a layer of activated glial cells that do not exhibit the same neurochemical responsiveness of normal, healthy brain tissue, Figures 1B,C (Jaquins-Gerstl et al., 2011). Microdialysis probe tracks are surrounded by glia exhibiting marked hyperplasia and hypertrophia, Figure 1B. A closer view, Figure 1C, shows that the track is encircled around most of its circumference (∼75%) by a barrier of GFAP immunoreactive elements, revealing that glial encapsulation and isolation of the probe is underway 24 h after implantation. The field of view in Figure 1C prominently displays a glial cell extending a process in excess of 300 μm in a linear fashion from the cell body toward the probe track.

We are not the first to report the penetration injury associated with microdialysis probes. Drew’s work examined the tissue surrounding microdialysis probes implanted in the striatum by light microscopy and revealed tissue damage 1.4 mm and neuronal loss 400 μm from the probe track (Clapp-Lilly et al., 1999). Drew also used hibernation as a model of neuroprotection by placing microdialysis probes into the striatum of the Arctic ground squirrel. Activated microglia and astrocytes were dramatically attenuated around the probe tracks in hibernating animals compared to euthermic controls (Zhou et al., 2001). Not only does the large size of the microdialysis probe cause damage, but the tissue response to the probe also contributes to the severity of the injury.

Implantation results in activation of biochemical and cellular mechanisms to heal the injury. The initial response is initiated as soon as a device is implanted. It may last from minutes to weeks depending on the injury (Szarowski et al., 2003). A host of immune processes are set into motion which have been well-documented in current literature. Following penetration injury from the microdialysis probe, the blood brain barrier is compromised due to vascular damage, and blood-borne macrophages enter the brain and become indistinguishable from resident microglia. Microglia/macrophages then transform morphologically and functionally into an active state, home toward the site of injury, and are involved in phagocytosis and debris clearance (Stence et al., 2001; Kozai et al., 2012, 2015, 2016, 2017; Bettinger et al., 2020). In addition, microglia and blood-borne macrophages can release reactive oxygen species, which can damage healthy bystander cells such as neurons.

**FIGURE 2:**
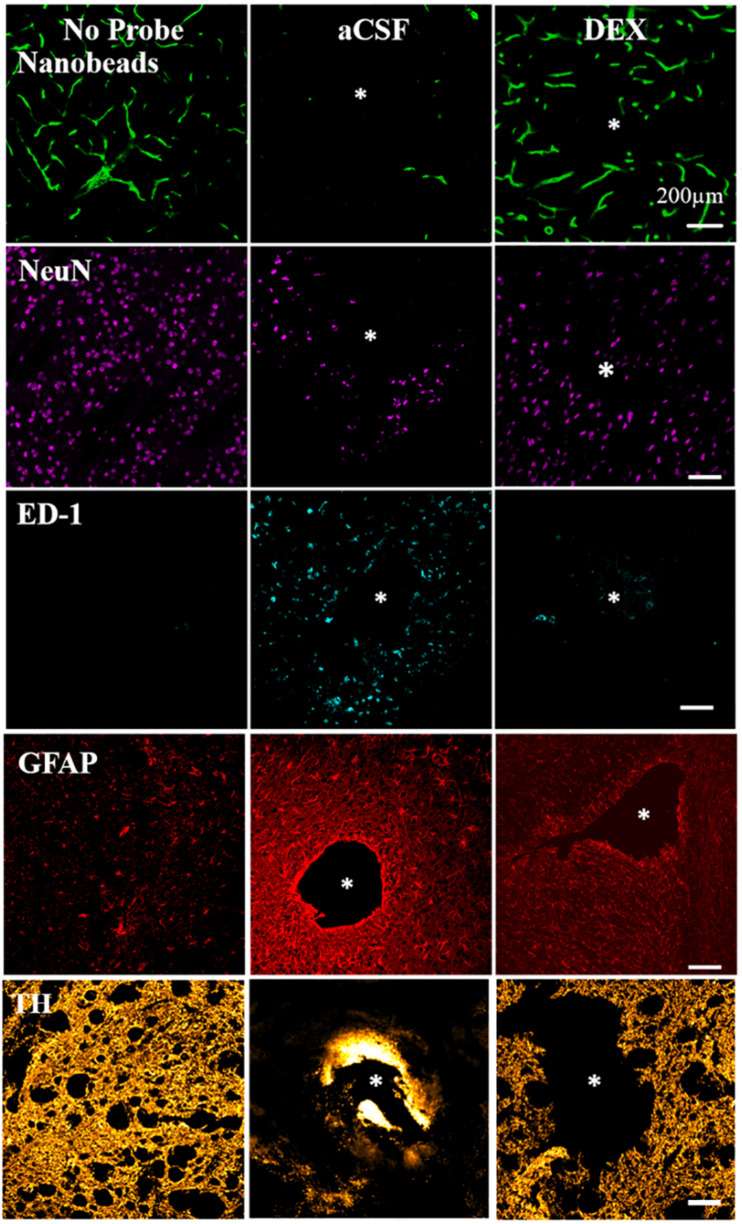
DEX mitigates the histochemical impact of penetration injury. Separate columns provide representative images of tissue after retrodialysis of aCSF, and DEX for 4 h. The left-most column shows images of non-implanted control striatal tissue. Separate rows provide representative images of tissue labeled with markers for blood flow (nanobeads), neuronal nuclei (NeuN), macrophages (ED1), glial fibrillary acidic protein (GFAP), and dopamine axons and terminals (TH). The probe track is in the center of the asterisk. Scale bar = 200 μm. Adopted from reference (Jaquins-Gerstl and Michael, 2009).

From our own studies we know that microdialysis probe implantation causes inflammation: it restricts blood flow (ischemia), activates macrophages, reduces neurons, diminished dopamine terminal, and axons and triggers gliosis, shown in Figure 2 (Jaquins-Gerstl and Michael, 2009; Nesbitt et al., 2013). Therefore, the tissue sampled by microdialysis is not in its normal state. Even though the injury associated with microdialysis probe implantation is significant, it is important to note that overall brain function and animal behavior does not change (Jaquins-Gerstl and Michael, 2009; Nesbitt et al., 2013). Efforts to reduce the tissue response associated with neural device implantation have been made and are further discussed in this manuscript.

### Strategies to Reduce Inflammation

Inflammation is a complex reaction involving protein adsorption, leukocyte migration, localization and activation, and secretion of inflammatory mediators (Ghirnikar et al., 1998; Lefkowitz and Lefkowitz, 2001; Zhong and Bellamkonda, 2005; Klueh et al., 2007; Anderson et al., 2008; Winslow and Tresco, 2010; Chao et al., 2012). The degree of intensity of this response is largely influenced by the extent of tissue injury, implantation site, implant shape and size, and chemical and physical properties of the membrane material (Bridges and García, 2008). Strategies to mediate inflammation are a subject of much interest. It is thought that preventing non-specific protein adsorption and subsequent immune cell adhesion onto the biomaterial surface (Wisniewski and Reichert, 2000; Wisniewski et al., 2000; Bridges and García, 2008; Helmy et al., 2009) can reduce leukocyte recruitment and tissue fibrosis (Gerritsen et al., 1999; Gifford et al., 2006). Although this has proven successful *in vitro*, implementing these *in vivo* have not translated into decreasing the inflammatory responses (Wisniewski et al., 2001; Polikov et al., 2005).

A more direct and active approach regulating the tissue response has been through the delivery of anti-inflammatory agents (Nakase et al., 2002; Zhong and Bellamkonda, 2005, 2007; Kim et al., 2007; Klueh et al., 2007; Nichols et al., 2012; Wang et al., 2012; Zachman et al., 2012; Koh et al., 2013). One such anti-inflammatory agent used in this area is glucocorticoid steroids. Glucocorticoid steroids are a class of steroids that bind to the glucocorticoid receptor which regulates inflammation; they have been used extensively to treat inflammatory conditions (Chao et al., 2012; Webber et al., 2012).

Dexamethasone is a potent synthetic glucocorticoid associated with diminished migration and activation of immune cells, upregulation of anti-inflammatory cytokines, and decreased collagen production at the implant site (Schmidt et al., 1999; Hickey et al., 2002; Norton et al., 2005; Klueh et al., 2007; Chao et al., 2012). Once DEX crosses the cell membrane it binds to specific cytoplasmic receptors such as the glucocorticoid receptors and then moves into the nucleus (Patacsil et al., 2016). This ability to cross cell membranes results in interference of leukocyte infiltration at the inflammation site along with inhibiting other inflammatory mediators.

**FIGURE 3:**
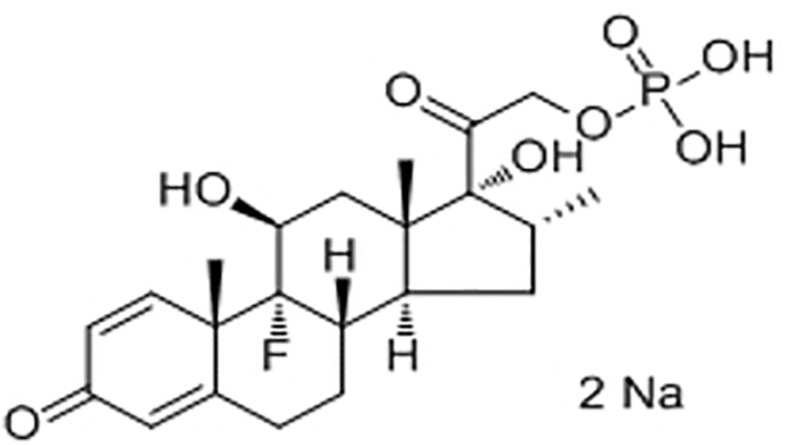
Chemical structure of dexamethasone 21-phosphate disodium.

Like many glucocorticoids, DEX has limited solubility. To overcome this problem, manufacturers have formulated DEX as water soluble hemisuccinate or phosphate ester pro-drugs. DEX 21-phosphate disodium (Figure 3), a phosphate ester pro-drug of DEX, is converted to active steroid DEX in blood rapidly and completely. DEX is used to treat many different inflammatory conditions such as allergic reactions, skin conditions, ulcerative colitis, arthritis, lupus, psoriasis, and breathing disorders. Most recently, it was used to treat patients hospitalized with COVID-19 (Lammers et al., 2020; Stauffer et al., 2020).

## Applications

Dexamethasone has a wide variety of uses in the medical and science field and is widely prescribed. This section discusses applications of DEX used in science as an anti-inflammatory drug, see Table 1 for a summary.

**TABLE 1:** Summary of DEX applications.

Author (year)	Dose	Treatment	Device type	Indwelling period	Comments
Shain et al., 2003	200 mg/kg	Peripheral injection once	Neural prosthetic	1 day, 1 and 6 weeks	Reduced gliosis near the implanted
Spataro et al., 2005	200 mg/kg	Subcutaneous injections daily and single	Neural prosthetic	1 and 6 weeks	Attenuated astroglia while microglia and vascular responses increased
Zhong and Bellamkonda, 2007	Nitrocellulose-DEX (100 μg) coating, thickness = 1.72 μm	Coating, continuous	Neural prosthetic	1 and 4 weeks	Attenuate the inflammatory response and reduce neuronal loss
Keeler et al., 2015a	20 μg/ml DEX	Infusions performed in 1-h increments for 6 h, in subcutaneous space near the spine	Microdialysis probe	7 days	Delayed infusion resulted in an increased percentage of CD68^+^, CD163^+^, and an up regulation in IL-6, decrease in CCL2 concentrations, macrophages shift to a M(DEX) activation state
Keeler et al., 2015b	20 μg/ml DEX	Infusions performed in 1-h increments for 6 h, dorsal subcutaneous space	Microdialysis probe	3 days	Decreases in both IL-6 and CCL2 in non-delayed DEX, increase in IL-6 in delayed DEX, macrophages shift to a M(DEX) activation state
Nesbitt, 2015	10 μM for 24 h then 2 μM DEX (less than 20 nanomoles)	Retrodialysis in striatum	Microdialysis probe	4 and 24 h	Stabilizes evoked dopamine responses and protects DA terminals
Kozai et al., 2016	10 μM DEX	Retrodialysis in cortex	Microdialysis probe	6 h	Significantly reduces microglial activation, T-stage morphology, and directionality polarization
Varner et al., 2016	10 μM for 24 h then 2 μM DEX (less than 20 nanomoles)	Retrodialysis in striatum	Microdialysis probe	5 days	Reinstated evoked dopamine activity, suppresses activation of microglia
Varner et al., 2017	10 μM for 24 h then 2 μM DEX (less than 20 nanomoles)	Retrodialysis in prefrontal cortex	Microdialysis probe	2 h, 5 and 10 days	Improved the detection of spreading depolarizations, induced transients and increased amplitudes of K^+^ spikes
Robbins et al., 2019	10 μM for 24 h then 2 μM DEX	Retrodialysis in prefrontal cortex with/out CCI	Microdialysis probe	10–12 days	Facilitates the monitoring of spontaneous spreading depolarizations

### DEX and Neuronal Devices

Several studies have examined the use of DEX with regard to neural prosthetics. In 2003, Shain et al. (2003) published a study using DEX to reduce gliosis near implanted silicone devices in the brain. Peripheral injections of DEX were made by subcutaneous injection. DEX was dissolved in ethanol (0.2 mg/mL) and delivered to produce a dose of 200 mg/kg. This study revealed that DEX reduced gliosis near the implanted device. However, the effects of the drug on the major inflammatory cells at the interface, which were macrophages, were not investigated.

Later in 2005, Spataro et al. (2005) injected DEX (200 mg/kg) for 1 or 6 days subcutaneously and showed the tissue reaction around neural implants was reduced. DEX treatment greatly attenuated astroglia responses. Cohorts with 6-day treatment showed this was more effective than a single injection regime.

Others have used DEX (100 μg) incorporated into a nitrocellulose coating deposited on electrodes for local drug delivery (Zhong and Bellamkonda, 2007). The local delivery of DEX reduced inflammation at 1-week post implantation but not at 4 weeks (Zhong and Bellamkonda, 2007), possibly because the amount of drug incorporated into the coating was not high enough for long-term release at a sufficient dosage.

Dexamethasone has been used to down regulate nitric oxide production which protects neurons (Zha et al., 2011), (Hempen et al., 2002), inhibits astrocyte proliferation and inhibited proliferation of NG2 cells (Kim and Martin, 2006). Because DEX can have serious side effects other studies have incorporated DEX into different probe coatings; poly(ethyl-vinyl) acetate, nitrocellulose, carbon nanotubes, and poly (lactic-co-glycolic acid) nanoparticles within alginate hydrogel matrices (Hempen et al., 2002; Kim and Martin, 2006; Zhong and Bellamkonda, 2007; Mercanzini et al., 2010; Luo et al., 2011). Benefits common to all the studies included decreased astrocytic response, reduced microglial/macrophage activity, mitigated neuronal loss, and minimized chondroitin sulfate proteoglycan expression.

### Dopamine Microdialysis and Detection With Fast Scan Cyclic Voltammetry

Dopamine (DA) is one of the analytes commonly sampled with microdialysis. DA is an important neurotransmitter with numerous roles in normal brain function and it is also involved in a variety of disorders including substance abuse, schizophrenia, and Parkinson’s disease (Lotharius and Brundin, 2002; Phillips et al., 2003; Schultz, 2007; Brisch et al., 2014). Microdialysis coupled to fast scan cyclic voltammetry (FSCV) is a method commonly used in our lab and many others for studying DA *in vivo* (Garris and Wightman, 1995; Robinson et al., 2003; Michael and Borland, 2006).

Previous studies from our lab demonstrate that the penetration trauma associated with microdialysis profoundly changes DAergic activity near the probe site. We placed voltammetric microelectrodes in the tissue adjacent to the probes. We compared DA as measured with microelectrodes placed 1 mm from the probes, 235 μm from the probes, and immediately adjacent to the probes, called a dialytrode. These recording locations produce dramatically different results, revealing a previously unrecognized 1000-fold gradient of DA activity across the traumatized tissue layer. DA levels measured 1 mm from the probe were in the micromolar range, whereas DA levels 235 μm from the probes were in the nanomolar range and the DA response at the dialytrode was completely abolished (Borland et al., 2005; Yang and Michael, 2007). Only after the administration of a dopamine uptake inhibitor nomifensine, (20 mg/kg i.p.) was DA detectable at the dialytrode. We questioned “why does penetration trauma of the microdialysis probe have an extreme impact on *in vivo* measurements of DA?”

**FIGURE 4:**
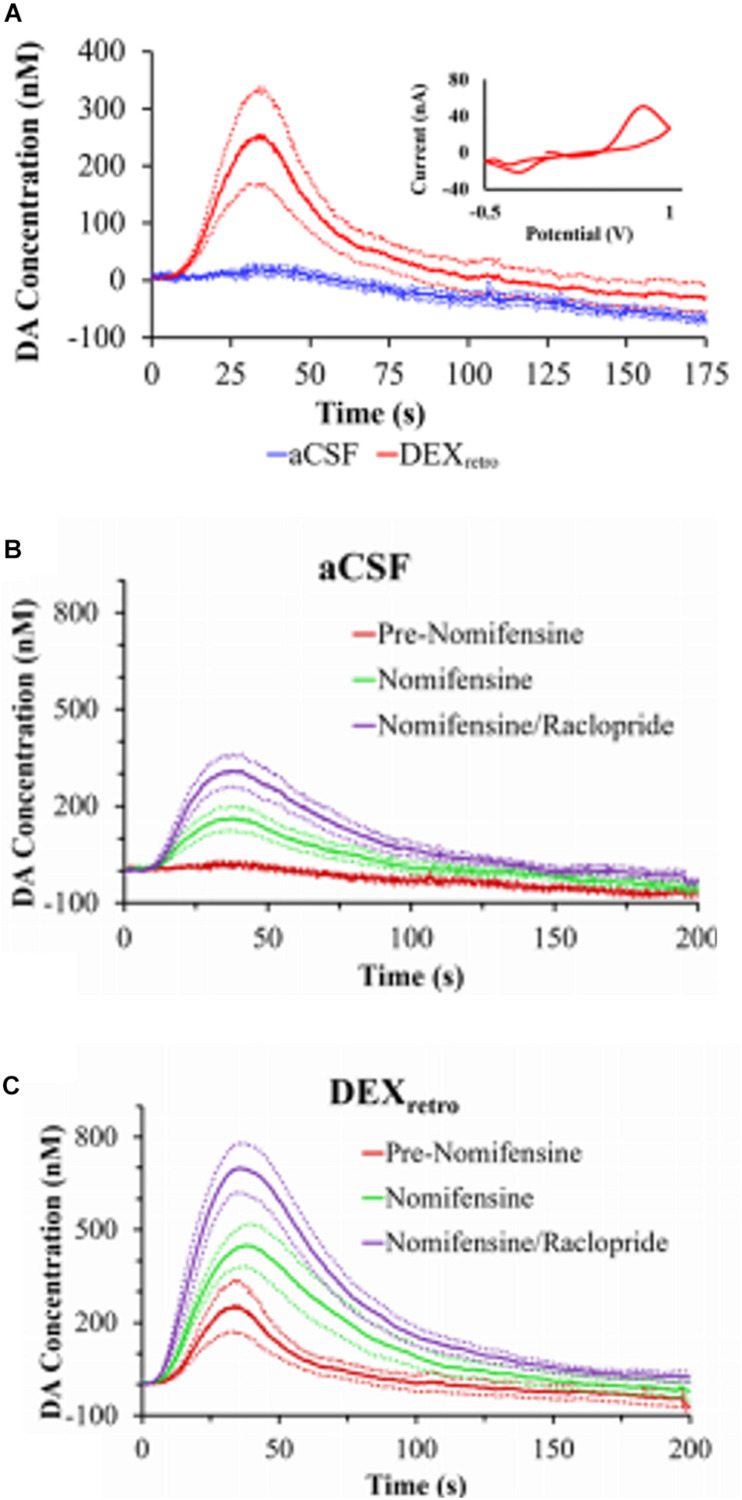
**(A)** Evoked DA responses (mean ± SEM) recorded at the outlet of microdialysis probes 5 days after implantation. Without DEX (blue) the stimulus evoked no response. With DEX (red), the stimulus evoked clear and reproducible responses. Inset: the average background-subtracted cyclic voltammogram obtained with DEX, showing the expected DA oxidation and reduction peaks. **(B)** Evoked DA responses (mean ± SEM) measured at the outlet of microdialysis probes perfused with aCSF or **(C)** DEX. DA was measured before nomifensine (red), after nomifensine (green), and then again after raclopride (purple). Adopted from reference (Varner et al., 2016).

One of our group’s objectives was to reduce, if not eliminate, the penetration injury and its deleterious effects on neurochemical monitoring. Motivated by the findings of Shain and others (Turner et al., 1999; Shain et al., 2003), we investigated the retrodialysis of DEX in the rat striatum. We showed that combining a 5-day post-implantation wait period with continuous retrodialysis of a low-micromolar concentration of DEX vastly reduces both the voltammetric and histological signs of the penetration injury, Figure 4 (Varner et al., 2016). DA measurements were taken at the outlet of the probe. At 5-days after probe implantation, retrodialysis of DEX: (1) reinstates normal evoked DA release activity in the tissue adjacent to the probe, (2) facilitates robust detection of evoked DA release, (3) establishes quantitative agreement between evoked DA measured simultaneously at the probe outlet and in the tissue next to the probe, (4) reinstates normal immunoreactivity for tyrosine hydroxylase and the dopamine transporter near the probe, and (5) prevents glial scarring at the probe track (Varner et al., 2016). Our findings support that the beneficial effects of DEX in this application may be attributed to its actions as an anti-inflammatory agent.

**FIGURE 5:**
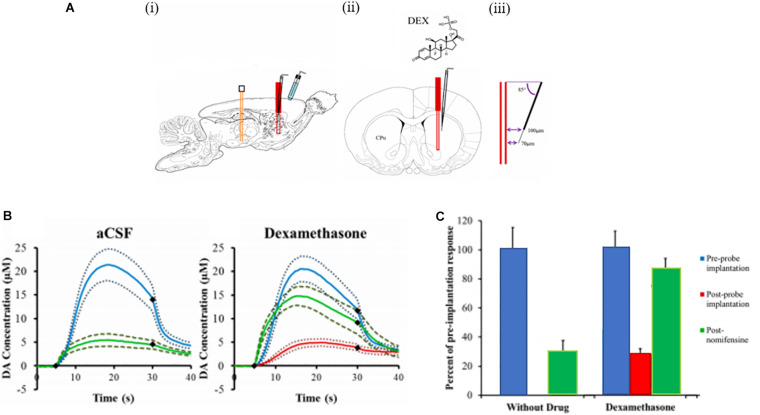
**(A)** A schematic of the placement of the devices in the rat brain. (i) A sagittal view of the stimulating electrode (orange) in the MFB, the microelectrode (black) and the microdialysis probe (red) in the striatum (CPu), and the Ag/AgCl reference electrode (blue) in contact with the brain surface. (ii) A coronal view showing the microelectrode at a 5° angle from the probe. (iii) The tip of the carbon fiber is 70 μm from the probe and the top of the fiber is 100 μm from the probe. **(B)** The effect of aCSF and DEX on electrically evoked DA responses measured before implanting the probe (blue lines), 2 h and 40 min after implanting the probe (red lines, the response was non-detectable in aCSF, and 25 min after nomifensine (green lines). The solid lines are the average responses in each group of rats and the broken lines are confidence intervals based on the standard error of the mean of each data point. The black diamonds show when the stimulus begins and ends. **(C)** The amplitude of evoked DA responses in the rat striatum in the presence of probes perfused with aCSF and DEX. The response amplitudes observed after implanting the probes (red) and after nomifensine (green) are normalized with respect to the amplitude observed before implanting the probes (blue = 100%). The bars show the mean ± SEM of the normalized results. DEX significantly increased evoked DA after implanting the probe. Adapted from reference (Nesbitt et al., 2013).

We also used voltammetry next to microdialysis probes to record electrically evoked DA release during the retrodialysis of DEX (Nesbitt et al., 2013). In this study a carbon fiber voltammetric electrode was inserted into the striatum of an anesthetized rat and a stimulating electrode was lowered into the medial forebrain bundle (MFB), Figure 5 (Nesbitt et al., 2013). Electrically evoked release was recorded by FSCV during electrical stimulation of the MFB. The final position of the probe was such that the distance between the tip of the microelectrode and the surface of the probe was 70 μm and the distance between the top of the electrode and the probe was 100 μm. This investigation focused on acute implants only 4 h in duration. We observed that microdialysis probes disrupt evoked DA release. If microdialysis probes were perfused with no DEX, all the electrically evoked DA responses were abolished (Nesbitt et al., 2013). DA was not detected during any of the electrical stimuli applied after implanting the probe. Next, a dose of nomifensine (20 mg/kg i.p.) was given to the rats which caused stimulated DA release. In the case of microdialysis probes perfused with DEX, implanting the probe next to the microelectrode diminished, but did not abolish, electrically evoked DA release. Again, these results confirm that DEX preserved DA activity in the tissue next to the microdialysis probes.

Immunohistochemistry was performed on brain tissue containing the probe using markers for ischemia, neuronal nuclei, macrophages, and DA axons and terminals (tyrosine hydroxylase). Probes perfused with no DEX caused profound ischemia, a loss of striatal neurons near the probes, activation of macrophages and a loss of tyrosine hydroxylase meaning profoundly disrupted DA axons and terminals (Nesbitt, 2015). Tissue with DEX perfusion showed a decrease in ischemia, neurons near the probe and few activated macrophages, indicating that DEX protected the brain tissue near the probe. Tyrosine hydroxylase was preserved in tissue surrounding the probe with DEX. We conclude that retrodialysis of DEX mitigates penetration injury during brain microdialysis (Nesbitt, 2015).

Using a longer time frame, 4 and 24 h, we implanted microdialysis probes in the rat striatum. We used with and without DEX in the perfusion fluid and measured evoked DA release at the outlet of the probes with FSCV. Responses at the probe outlet were below the detection limits of FSCV unless animals were treated with nomifensine, which increases the microdialysis recovery of evoked dopamine transients (Church et al., 1987; Pontieri et al., 1995). When probes were perfused without DEX, post-nomifensine responses at the probe outlet exhibited a significant decline in amplitude between 4- and 24-h post-implantation. However, DEX abolished this instability, both in animals treated first with nomifensine and then with raclopride, a D_2_ dopamine receptor antagonist. This study demonstrated that DEX stabilizes, but does not alter, evoked DA responses at the outlet of microdialysis probes.

### Dexamethasone and Macrophage Polarization States

Understanding the biochemistry that occurs at the site of an implanted biomaterial is important in a wide range of clinical contexts. Julie Stenken’s group from the University of Arkansas has used microdialysis sampling with DEX to understand the inflammatory response caused by macrophages. Although DEX has widely been used as a releasing agent in biomaterials, Stenken and others have identified DEX as a modulator which produces a phenotype that has characteristics of the M2c macrophage (Van Coillie et al., 1999; Martinez, 2011; Keeler et al., 2015b). Macrophages play roles in opposing processes such as inflammation vs anti-inflammation and tissue destruction vs tissue remodeling. Macrophages are driven by micro-environmental chemical signals present within the extracellular matrix which result in different macrophage polarization states (Gordon and Taylor, 2005; Mantovani et al., 2013; Keeler et al., 2015b). While different materials have been used to elicit a desired macrophage activation state, it is unknown whether modulators can be used to shift the macrophage activation state. Stenken et al. was the first to use modulators to attempt regulating the macrophage activation state at an implant site. The primary hypothesis was that by producing a predominantly M2c activation state, improved healing would be seen at the implant site (Keeler et al., 2015b). In this study, DEX was delivered through the microdialysis probe to alter the activation state of macrophages in awake Sprague-Dawley rats. Two probes were implanted in the subcutaneous space on either side of the spine with ∼1-inch separation between the probes. The delivery of DEX (20 μg/ml) resulted in an increase in the percentage of M2c macrophages seen in the tissue surrounding the microdialysis probe. Remarkably, differences were seen when DEX was delivered immediately following probe implantation as compared to a delayed delivery. In tissue where DEX was immediately delivered, fewer macrophages were present compared to the delayed delivery.

The gene expression profiles of the chemokine CCL2 and the inflammatory cytokine IL-6 were also examined; these too were different. CCL2 is known to be one of the primary chemokines responsible for the migration and infiltration of monocytes to a wound site. Once at the wound site, monocytes differentiate to macrophages. While both CCL2 and IL-6 were significantly down-regulated in the tissue from immediate DEX delivery, IL-6 was seen to be significantly up-regulated in response to a delayed DEX infusion. These results showed that it is possible to use modulators to shift macrophages *in vivo* to a desired activation state at an implant site while also characterizing a predominantly M2c environment (Keeler et al., 2015b).

The time course required for altering macrophages was also examined by Stenken et al. by delivering DEX through an implanted microdialysis probe in the subcutaneous space of male rats (Keeler et al., 2015a, b). They investigated a 3-day post-implantation time period for initiating DEX infusion. They sought to determine if the start of DEX infusion is delayed, allowing the initial inflammatory response to begin, would be more optimal in terms of converting macrophages to an M(Dex) state. The resulting foreign body response to the implanted microdialysis probes was examined by immunohistochemical and molecular means at the gene and protein level. The delayed delivery of DEX resulted in an upregulation of IL-6 gene transcripts as well as a moderate decrease in CCL2 concentrations (Keeler et al., 2015a, b). The delayed DEX treatment resulted in an increase in cellular density in the tissue surrounding the microdialysis probe. More importantly the delayed delivery of DEX shifted the macrophages to an M(Dex) activation state. Most studies involved in the use of modulators to shift the activation state of macrophages has primarily been *in vitro*. Stenken’ s work is cutting edge as it demonstrates the use of microdialysis sampling to deliver DEX to alter macrophage polarization *in vivo*, which improves tissue remodeling.

### DEX Retrodialysis Attenuates Microglial Activity

Microglia play a critical role in living brain tissue. They perform a wide range of tasks while in the native ramified state; they are constantly scavenging the CNS for plaques, damaged neurons, and synapses. They are also involved in experience-dependent synaptic maintenance (Wake et al., 2009; Tremblay et al., 2010), debris clearing (Nimmerjahn et al., 2005), and surveillance against injury and invasion (Nimmerjahn et al., 2005). Microglial cells are extremely sensitive to even small pathological changes in the CNS and are also extremely plastic. They adopt a specific form in response to the local conditions and chemical signals they have detected (Nimmerjahn et al., 2005). When a brain insult is detected by microglial cells, they enter an ameboid form called a transition stage (*T*-stage); this process is generally referred to as “microglial activation.” During this stage, the resting microglia retract their processes, which become fewer and much thicker, increase the size of their cell bodies, change the expression of various enzymes and receptors, and begin to produce immune response molecules. Acutely, following probe insertion, nearby microglia activate and encapsulate the implant with their processes and lamellipodia sheath (Kozai et al., 2012).

**FIGURE 6:**
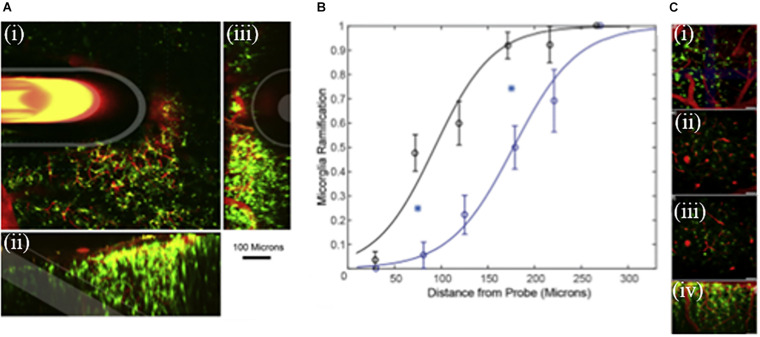
**(A)** Microdialysis probe *in vivo* into the cortex. Green represent microglia and red blood vessels. (i) 100 μm thick Z-projection of a microdialysis probe implanted into the tissue. The image is parallel to the skull. Microdialysis, Outer membrane and fused silica tube is highlighted in white. (ii) Side view 3D reconstruction of microdialysis probe. (iii) 3D reconstruction of the tissue rotated; probe is projecting out of the image. Scale = 100 μm. **(B)** Curve characterizes microglia ramification vs the distance from the probe. Index = 1 represents all microglial cells being ramified, while index = 0 represents all microglial cells being activated around the microdialysis probe perfused with Dex (black, *n* = 8) and aCSF (blue, *n* = 8). Line is the best fit logarithmic binomial generalized linear regression curve. Cyan stars indicate significant difference (*p* < 0.05). **(C)** Zoomed in images (i–iv) of ramified microglia. Adapted from reference (Kozai et al., 2012).

We wanted to understand how DEX affects microglia morphology/motility in real time by characterizing the dynamic microglia response to penetration trauma of microdialysis probes. We employed *in vivo* two-photon microscopy to quantify the acute microglial response to microdialysis probes in the brain with or without retrodialysis of DEX. We examined the cellular microglial response to microdialysis probe insertion up to 6 h; morphological changes and activation characteristics of microglia around the implants were observed and quantified, Figure 6 (Kozai et al., 2016). We found that implantation of the microdialysis probe with DEX reduced microglial activation. In tissue without DEX (aCSF perfused) the activation state of the microglia were delayed and were significant. DEX had a significant effect on the radius of microglia activation, morphology, *T*-stage activation, and microglia directionality index. The temporal dynamics of microglial response also showed distinct differences between the control and DEX treated tissue. While many microglia appeared non-polarized with unusually thicker processes in the DEX treated tissue at 6 h, some polarized toward nearby blood brain barrier. In this study the local administration of DEX rendered it a great candidate to reduce the effects of penetration injury by neural probes (Kozai et al., 2016).

### Spreading Depolarizations and Microdialysis

One application of clinical microdialysis is monitoring brain injured patients in the intensive care unit to identify chemical markers of secondary brain injuries. Our group and others have focused on a phenomenon of secondary injury called spreading depolarization (SD). Incidences of SDs are significantly correlated with poor patient outcomes, including death, vegetative state, and severe disability (Hartings et al., 2009, 2011a, 2011b; Lauritzen et al., 2011). SD is characterized by a wave of near-complete depolarization of neurons and glia resulting in a temporary disruption of the ion homeostasis and silencing of electrical activity (Guiou et al., 2005; Fabricius et al., 2006; Dreier et al., 2006a, b, 2009; Strong et al., 2007; Dreier, 2011; Seule et al., 2015; Rogers et al., 2017). Excess K^+^ released into the extracellular space during SDs can diffuse to and depolarize neighboring cells, thus creating a wave that propagates across the cortex at a rate of 2–5 mm/min. The brain tissue depends on the vasculature to deliver glucose and oxygen to meet these energy demands. The swelling and restricted blood flow commonly observed after a TBI can hinder this process. Clusters of SDs impose particularly severe energy demands on the injured brain and can result in long-lasting declines in basal glucose (Fabricius et al., 2006; Dreier et al., 2006a; Feuerstein et al., 2010; Nakamura et al., 2010; Lauritzen et al., 2011; Hartings et al., 2011a, b).

**FIGURE 7:**
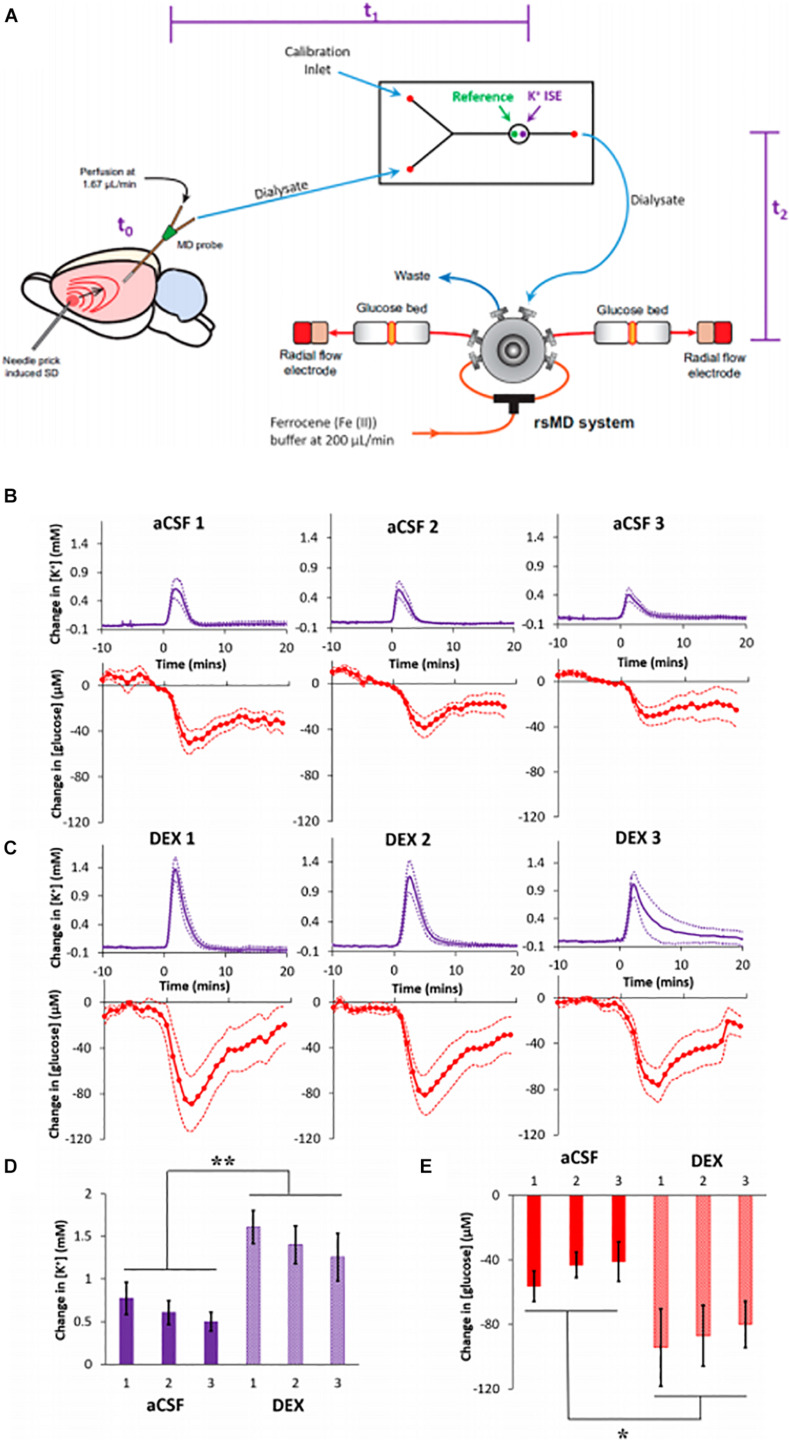
**(A)** Experimental design of the rsMD. SD was induced by needle pricks in the cortex. The SD arrives at the microdialysis probe at t0: intervals between the needle pricks and t0 were typically less than 1 min. Next, the sample travels to the K + ISE in approximately 4 min, t1. Finally, the sample travels to the glucose detector in approximately 7 min, t2. **(B)** Cortical responses to three needle pricks recorded 2 h after probe insertion with aCSF or **(C)** DEX (mean ± SEM). Maximum changes in **(D)** K + and **(E)** glucose were analyzed with two-way ANOVAs with group (aCSF, DEX) and needle prick (1, 2, 3; repeated measures) as the factors. The needle prick and interactions were not significant, but group was significant for both K + [F(1,14) = 13.422] and glucose [F(1,14) = 6.253]. **p < 0.005 and *p < 0.05. Adapted from reference (Varner et al., 2017).

The importance of SD monitoring during neurointensive care has been widely recognized and numerous animal studies have been dedicated to improving SD monitoring (Dreier, 2011; Ayata and Lauritzen, 2015; Dreier et al., 2016; Osier and Dixon, 2016). Methods for SD monitoring include electrocortiograph (ECoG), blood flow analysis, and microdialysis. The Boutelle lab has developed a rapid sampling microdialysis (rsMD) system (Figure 7) that monitors SD-associated changes in glucose and lactate at the patient’s bedside (Jones et al., 2002). Combining the rsMD system with an online K^+^ ion-selective electrode and ECoG provides a multimodal analysis system that can detect episodes of SDs in the days following the patient’s primary injury (Rogers et al., 2013, 2017; Papadimitriou et al., 2016).

Our group reported that DEX conferred profound benefits to the microdialysis monitoring of SDs induced glucose and K^+^ transients in the rat cortex, Figure 7 (Varner et al., 2017). We inserted microdialysis probes, with and without retrodialysis of DEX, and monitored SD-induced glucose and K^+^ transients 2 h, 5 days, or 10 days later (Varner et al., 2017). SDs were induced by needle pricks and were performed at 30-min intervals. Retrodialysis of DEX improved the detection of SD-induced transients at all three time points. DEX increased the amplitudes of the SD-induced K^+^ spikes and glucose dips by 127% and 86% (averages of the three responses), respectively, compared to those observed without DEX. In the presence of DEX, the amplitudes of the K^+^ spikes were significantly larger at 2 h compared to 5 and 10 days. In contrast, there were no significant differences between the amplitudes of the glucose dips at the three time points. In the presence of DEX, the fraction of K^+^ spikes accompanied by quantifiable glucose dips was relatively constant across the three time points. In the absence of DEX, glucose dips were essentially non-detectable 5 days after probe insertion.

In our 10-day studies, DEX retrodialysis was performed only during the first 5 days, confirming that continuous DEX delivery for the entire 10-day time window is not required. After retrodialysis of DEX, histochemical inspection of probe tracks found no signs of ischemia or gliosis 10 days after insertion (Varner et al., 2017). Our findings confirmed that DEX enhances the performance of microdialysis for monitoring SD-induced glucose and K^+^ transients in the rat cortex for at least 10 days after probe insertion.

Our most recent study was to investigate whether the microdialysis with DEX is translatable to the monitoring of spontaneous SD in rats after controlled cortical impact (CCI), a widely studied rodent model of TBI (Dixon et al., 1987; Wagner et al., 2005; Xiong et al., 2013). Microdialysis probes were placed 1 or 3 mm away from the CCI site expecting that the secondary injury would spread over time into the penumbra (Robbins et al., 2019). But this did not happen. There was no difference between 1 and 3 mm location of the probes. We recorded K^+^ and glucose in dialysates from 10 rats for 10–12 days following CCI and microdialysis probe insertion (Robbins et al., 2019). Overall, recordings from *n* = 8 rats exhibited 185 spontaneous SDs, hallmarked by a transient rise, and fall in dialysate K^+^, over the course of 5–7 min. Of the SDs observed while we were also monitoring glucose (*n* = 126), some were accompanied by negative glucose transients (*n* = 89), no obvious change in glucose (*n* = 25), or transient increases in glucose (*n* = 12). Some rats (*n* = 2) exhibited no SDs, which seems to be consistent with clinical reports that SDs are detected in some but not all TBI patients who undergo neuromonitoring (Hartings et al., 2011a, b; Dreier et al., 2016).

We also observed a second post-CCI phenomenon consisting of a slow, progressive decline of dialysate glucose from basal concentrations to levels below the detection limit (Robbins et al., 2019). Once this occurred, glucose concentrations did not return to detectable levels. We found a high degree of animal-to-animal variability in the outcome of chemical monitoring after CCI. Individual animals varied widely in the number and frequency of SDs and the onset and duration of progressive glucose decline. We attribute this to the varied extent of the injury induced by CCI. Despite the animal-to-animal variability, this study yielded several consistent observations. Most rats (8 of 10) exhibited spontaneous SDs, either isolated or in clusters. Spontaneous SDs occurred before, during, and after the glucose decline.

Probe tracks were examined by immunohistochemistry and showed no presence of a glial barrier, absence of blood flow, or profound losses of neurons: these observations indicate the anti-inflammatory efficacy of DEX in the presence of CCI. This study adds to a mounting body of evidence that DEX-enhanced microdialysis facilitates extended intracranial microdialysis in the rat brain over the course of at least 10 days.

## Outlook

The development of long-term microdialysis is an inspiring yet greatly challenging process. The foreign bodies response toward the penetration trauma associated with the implant is a critical barrier to overcome even with the use of an anti-inflammatory drug such as DEX. We have shown studies where DEX-enhanced microdialysis was used to stabilize the surrounding tissue allowing for better detection of (a) DA, (b) attenuated microglia, (c) reduce gliosis, and (d) enhances the detection of K^+^ and glucose transients induced by spreading depolarization. DEX improves and stabilizes the tissue surrounding the probe and promotes longevity of the probe.

Current and cutting-edge research is being performed in clinical studies using DEX and microdialysis with the eventual goal of better patient outcomes. There are many types of cells involved in the bodies’ response to the microdialysis implant, although DEX is very successful at damping the immune response. Many questions still arise. To what extent does macrophages traffic across the blood brain barrier following chronic implantation? What is the extent of changes to the local vasculature and the intact blood brain barrier? Are there better strategies for reducing the foreign body response? Are there better suited drugs or probe coating which could be used with microdialysis? Understanding the penetration injury associated with probe implantation and providing protective strategies promotes long-term sampling by microdialysis and plays a lasting role in understanding the neurochemistry of the brain. The future of long term microdialysis is unlimited.

## Author Contributions

AJ-G and AM conceived the overall topics of discussion. AJ-G wrote the sections. Both authors read and approved the final manuscript.

## Conflict of Interest

The authors declare that the research was conducted in the absence of any commercial or financial relationships that could be construed as a potential conflict of interest.
